# Genomic analysis reveals *Lactobacillus sanfranciscensis* as stable element in traditional sourdoughs

**DOI:** 10.1186/1475-2859-10-S1-S6

**Published:** 2011-08-30

**Authors:** Rudi F Vogel, Melanie Pavlovic, Matthias A Ehrmann, Arnim Wiezer, Heiko Liesegang, Stefanie Offschanka, Sonja Voget, Angel Angelov, Georg Böcker, Wolfgang Liebl

**Affiliations:** 1 Lehrstuhl für Technische Mikrobiologie, Germany; 2 Lehrstuhl für Mikrobiologie, Technische Universität München, 85350 Freising, Germany; 3 Laboratorium für Genomanalyse, Institut für Mikrobiologie und Genetik, Georg-August-Universität Göttingen, Germany; 4 Ernst Böcker GmbH & Co KG, Minden, Germany

## Abstract

Sourdough has played a significant role in human nutrition and culture for thousands of years and is still of eminent importance for human diet and the bakery industry. *Lactobacillus sanfranciscensis* is the predominant key bacterium in traditionally fermented sourdoughs.

The genome of *L. sanfranciscensis* TMW 1.1304 isolated from an industrial sourdough fermentation was sequenced with a combined Sanger/454-pyrosequencing approach followed by gap closing by walking on fosmids. The sequencing data revealed a circular chromosomal sequence of 1,298,316 bp and two additional plasmids, pLS1 and pLS2, with sizes of 58,739 bp and 18,715 bp, which are predicted to encode 1,437, 63 and 19 orfs, respectively. The overall GC content of the chromosome is 34.71%. Several specific features appear to contribute to the ability of *L. sanfranciscensis* to outcompete other bacteria in the fermentation. *L. sanfranciscensis* contains the smallest genome within the lactobacilli and the highest density of ribosomal RNA operons per Mbp genome among all known genomes of free-living bacteria, which is important for the rapid growth characteristics of the organism. A high frequency of gene inactivation and elimination indicates a process of reductive evolution. The biosynthetic capacity for amino acids scarcely availably in cereals and exopolysaccharides reveal the molecular basis for an autochtonous sourdough organism with potential for further exploitation in functional foods. The presence of two CRISPR/cas loci *versus* a high number of transposable elements suggests recalcitrance to gene intrusion and high intrinsic genome plasticity.

## Background

**Figure 1:**
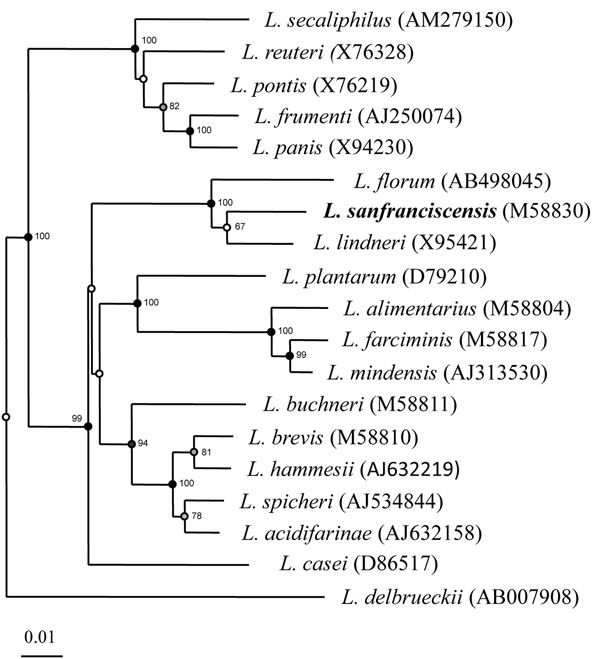
Neighbour joining phylogenetic tree of *Lactobacillus* species showing the phylogenetic position of *Lactobacillus sanfranciscensis* based on 16S rRNA gene sequences. The scale bar indicates 1 nucleotide substitution per 100 nucleotides. Numbers in parentheses indicate accession numbers of 16S rRNA genes from type strains. Bootstrap values over 50% (based on 100 replications) are shown at the noodes.

The use of sourdough is documented for > 5,000 years and of eminent industrial importance in the production of baked goods amounting to more than 3 million tons of baked goods annually [1]. Annual per capita consumption of baked goods in Europe is 50-85 kg with up to 20% involving sourdough fermentations with wheat or rye, i. e. a total of > 3 million tons. To date, no bacterial genomes from strains adapted to this huge man made habitat in millions of generations are available. *Lactobacillus sanfranciscensis* was first described in 1971 by Kline and Sugihara who isolated and characterized obligately heterofermentative lactobacilli from San Francisco sourdoughs [2]. The name *Lactobacillus sanfrancisco* refers to the city where the sourdoughs from which the organism was isolated had been propagated for more than 100 years. At that time the species was not included in the Approved Lists of Bacterial Names and had no standing in bacteriological nomenclature until the name was revived by Weiss and Schillinger in 1984 [3]. To follow the Rules of the International Code of Nomenclature of Prokaryotes the species epithet was changed to *L. sanfranciscensis*[4]. Sourdough fermentations worldwide are characterized by a highly stable association of yeasts and lactic acid bacteria. In rye and wheat sourdoughs with a tradition of continuous propagation by back-slopping procedures, *L. sanfranciscensis* is the probably the most adapted species and regarded as autochthonous key organism of the sourdough microbiota [5,6]. Its phylogenetic position within the genus lactobacillus is shown in Figure 1. Multiple metabolic activities of *L. sanfranciscensis* have been described in the literature that contribute to the quality of sourdough and baked goods. With the exception of one report by Groeneveld *et al*. [7], who allotted isolates from fruit flies exhibiting 97% rDNA sequence homologies as *L. sanfranciscensis*, this species has only been isolated from sourdoughs, while strains of all other species found in sourdough are frequently isolated also from other habitats. None of the genome-sequenced strains of these genera, e. g. *L. plantarum* or *L. reuteri* were isolated from sourdough. This raises the question for the role of man in evolution of *L. sanfranciscensis*.

The sourdough microbiota including *L. sanfranciscensis* contribute to dough rheology and flavour properties due to a strong acidification by an optimized carbohydrate metabolism and the liberation of precursors of volatile compounds by the proteolytic system [8-10] and the catabolism of specific amino acids [5,10,11]. Formation of exopolysaccharides (homopolysaccharides and fructooligosaccharides) enhance texture, shelf life and nutritional value [12]. The sequenced strain *L. sanfranciscensis* TMW 1.1304 was isolated in 2006 from a commercial mother sponge with a tradition of continuous propagation. The presence of this strain in this sourdough starter was demonstrated over a period of at least 20 years, and it accounts for more than 90% of the microflora of that product.

## Results and discussion

### General genomic features

The *L. sanfranciscensis* TMW 1.1304 genome project allowed the assembly of a circular chromosomal sequence of 1,298,316 bp and two additional plasmids of 58,739 bp and 18,715 bp. The genome size of strain TMW 1.1304 was estimated at approximately 1.3 Mbp on the basis of results obtained by pulsed-field gel electrophoresis of chromosomal DNA restriction fragments. A previous estimation of the apparent genome size based on four strains of *L. sanfranciscensis* isolated from Italian sourdoughs and the type strain DSM 20451^T^ indicates 1.4 Mbp [13]. Thus, the *L. sanfranciscensis* genome is the smallest genome within the genus *Lactobacillus* so far followed by the recently published genome of *Lactobacillus iners* AB-1 with 1,304 Mbp [14]. The general features of the sequence are presented in Table 1. The average orf length is 835 and the codon density is 87.1, which is in the range of other lactobacilli (Additional file 1).

**Table 1:** General features of the *L. sanfranciscensis* genome compared with genomes of other species, which are found in sourdough (however, the strains whose genomes have been sequenced were isolated from other sources). Data are from this study and [59].

	*L. sanfranciscensis* TMW 1.1304	*L. reuteri* JCM 1112^T^	*L. fermentum* IFO 3956
Chromosome size (bp)	1,298,316	2,039,414	2,098,685
Plasmid(s)	58,739 and 18,715	-	-
GC content (%)	34.7 (37.6 and 36,1)*	38.9	51.5
Total ORFs	1,437 (63 + 19)	1,820	1,844
Functionally assigned	791	1,211	1,212
Conserved hypothetical	498	413	360
Non-conserved hypothetical	148	196	272
Coding density (%)	88.1	83.6	80.4
tRNAs	61	58	54
rRNA operons	7	6	5
Phage-related ORFs	not detected	53	24
Transposases	111	55	106
Group II introns	not detected	12	0

* values in brackets are for the plasmids pLS1 and pLS2, respectively.

**Figure 2:**
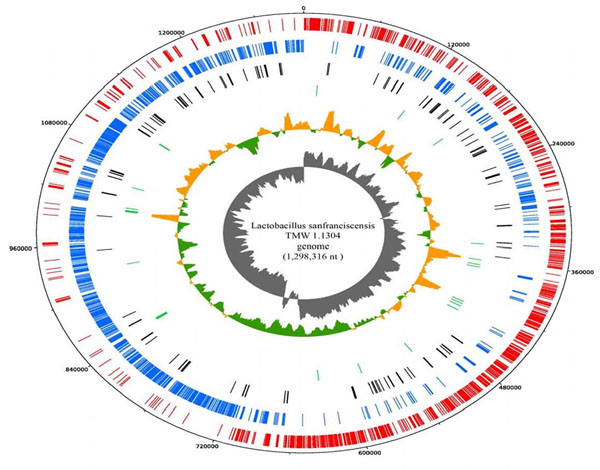
Genomic atlas of *L. sanfranciscensis* TMW 1.1304. From the outer circle inward, CDS on the forward strand (red), CDS on the reverse strand (blue), Pseudogenes on both strands (black), tRNA genes (green), GC content deviations from the average GC-content (green, low GC spike and orange, high GC spike) , GC skew (grey). The GC% and GC skew (C-G)/(C+G) were calculated in a window of 10000 nt, in steps of 200 nt.

On the basis of analysis of the GC skew (G-C/G+C), the cumulative GC-skew and the location of characteristic genes (chromosomal replication initiator protein DnaA) we could identify a typical bacterial origin of replication and its beginning was assigned as base-pair one of the genome. Thus, two equal replication arms (replichores) were present and the locations of the predicted 1,437 coding sequences on the two strands correlated well with the direction of replication (Fig. 2). There were 153 pseudogenes found randomly distributed in the chromosome. Genes encoding replication functions overlap sequences with significant changes in GC skew indicating the location of the origin of replication (oriC). This region harbors the genes for the replication initiator protein (*dna*A), the beta subunit of DNA polymerase III (*dna*N), and the DNA gyrase subunits A and B (*gyr*A and *gyr*B). The arrangement of these genes, *dna*A-*dna*N-*rec*F-*gyr*B-*gyr*A, is similar to that found in other Gram-positive bacterial genomes studied so far. Five DnaA-box consensus sequences (TTATNCACA) were found upstream of *dna*A and three in the *dna*A-*dna*N intergenic region. Opposite of the genome between position 628,700 and 628,800 a second change in GC skew indicates the replication terminus.

### Stable RNA gene density and codon usage bias

Seven rRNA operons and 61 tRNA genes were detected, demonstrating an intriguingly high density of genes for stable RNAs in the genome of *L. sanfranciscensis* TMW 1.1304. Analysis of approx. 1000 complete genomes (Additional file 2) available in GenBank revealed that *L. sanfranciscensis* TMW 1.1304 has the highest rRNA operon density (5.39 per Mbp) among all known free-living organisms. The only genome with a higher rRNA gene density is that of *Candidatus* Carsonella ruddii PV (1 rRNA operon in its 0,159662 Mbp genome), an obligate insect endosymbiont not capable of autonomous growth whose status as a living organism is debatable [15] due to the lack of most replication, transcription and translation genes considered as essential for living cells. Interestingly, 50% of the top 20 species with the highest rRNA gene densities (rRNA gene densities above 2.9 per Mbp; species represented by more than one sequenced genome were only counted once; genomes from non-free-living bacteria with canditatus status were disregarded) were lactic acid bacteria, i. e. various *Lactobacillus* and *Streptococcus* species (Additional file 3).

Multiple rRNA operons, which are found in many prokaryotes, may be of importance to achieve high growth rates and to adapt rapidly to changing environmental conditions [16,17]. We postulate that the exceptionally high rRNA operon density on the *L. sanfranciscensis* genome allows the bacteria to respond quickly to favorable growth conditions in their sourdough environment, rapidly initiating fermentative metabolism and fast growth which in combination with their specifically adapted metabolism (see below) could help to out-compete other contaminating bacteria. This strategy may also hold true for other lactic acid bacteria with a high rRNA operon density such as *Lactobacillus delbrueckii* subsp. *bulgaricus* (second-ranking after *L. sanfranciscensis* with an rRNA operon density of 4.85/Mbp of strain ATCC BAA-365) which is one of the classical starter organisms responsible for rapid lactic fermentation during yoghurt production.

Numerous microbial genomes reveal a codon usage bias (CUB), i.e. a pronounced preference for a specific set of codons (named major codons), in genes whose products are required in large quantities, which improves translation efficiency of these genes and contributes to optimizing cell growth [18,19][20]. The *L. sanfranciscensis* genome revealed a relatively strong CUB. A closer look at the set of genes that are translationally optimized in this organism revealed that the top 80 hits expectedly contained many genes (44) encoding ribosomal proteins and translation factors (Additional file 4), but also with the exception of the ribulose-5-phosphate epimerase gene all genes for the formation of lactate, CO_2_ and ethanol via the phosphoketolase pathway, underscoring the importance of the efficient expression of this pathway for *L. sanfranciscensis*.

### Carbohydrate metabolism

Consistent with the classification of *L. sanfranciscensis* as a heterofermentative lactic acid bacterium (LAB) all genes required for the phosphoketolase pathway are present in the *L. sanfranciscensis* genome whereas no homologues to transaldolase or transketolase were found. *In silico* analyses revealed that the sequenced *L. sanfranciscensis* strain is likely to use maltose, fructose, ribose and gluconate as carbon sources (Additional file 5). Additionally, two copies of a transporter for arabinose were found (LSA_1450, LSA_1460) of which only one seems to be functional as LSA_1460 is truncated at the 3’ end. The presence of two genes for oligo-1,6-glucosidase (LSA_05810; LSA_01770) indicates the ability to hydrolyse α-1,6-D-glucosidic linkages in oligosaccharides produced from starch and glycogen (isomaltulose, isomaltotriose, panose and isomaltose). Except for maltose phosphorylase (LSA_01510) and a truncated alpha-glucosidase (LSA_05800) no additional ORFs for glycoside hydrolases were annotated.

Growth of *L. sanfranciscensis* with maltose as carbon source is generally accelerated when fructose, citrate or α-ketoglutarate are used as alternative electron acceptors. [21,22]. Several uptake systems for possible electron acceptors were present. Fructose can be transported by a fructose permease (LSA_2810) and reduced to mannitol using a mannitol-2-dehydrogenase (LSA_02820). Two genes for citrate-sodium symporters (LSA_08630 and LSA_13030) and one gene encoding a malate uniport protein (LSA_02110) suggest uptake of citrate and malate. Genes necessary to reduce citrate to lactate are also present (LSA_12980 and LSA_12990 for citrate lyase, LSA_13020 for oxaloacetate decarboxylase, while no homologue gene for a succinate dehydrogenase which is necessary for the utilization of malate as electron acceptor was found.

The use of α-KG as electron acceptor by conversion to 2-hydroxyglutarate by LAB was previously mentioned by Radler and Broehl, 1984[23]. Recently, reduction of α-ketoglutarate to 2-hydroxyglutarate was demonstrated in *L. sanfranciscensis* indicating that α-ketoglutarate was used preferably as electron acceptor and NADH-dependent hydroxyglutarate dehydrogenase activity was confirmed by enzymatic analysis of crude cell extracts of *L. sanfranciscensis*[*22*]. Several orfs with putative α–hydroxy acid dehydrogenase activity were present.

As expected, the genome of *L. sanfranciscensis* only encodes an incomplete citrate cycle as only genes for fumarate hydratase (LSA_12040), malate dehydrogenase (LSA_02100; LSA_04670) and citrate lyase (LSA_12980 and LSA_12990) are present.

### Pyruvate metabolism

Due to a frameshift in the pyruvate oxidase gene (EC 1.2.3.3/ LSA_00220) direct conversion of pyruvate to acetyl phosphate is not possible for *L. sanfranciscensis.* Therefore, the organism most likely converts pyruvate to acetate via lactate first and then generates acetyl phosphate from acetate. Enzymes required for redundant pathways like formate C-acetyltransferase (EC 2.3.1.54) or acetaldehyde dehydrogenase (EC 1.2.1.10) were not encoded by the *L. sanfranciscensis* genome but in lactobacilli with larger genomes like *L. plantarum* or *L. casei*. In spite of the presence of only a relative low number of pyruvate dissipating enzymes in the *L. sanfranciscensis* genome, a high degree of redundancy for lactate dehydrogenase (*ldh*) encoding genes was observed as at least three L-lactate dehydrogenases (LSA_09870, LSA_11450, LSA_13040) and three D-lactate dehydrogenases (LSA_00860, LSA_10990, LSA_12510) were found, among which LSA_11450 and LSA_12510 are pseudogenes. The presence of several copies of *ldh* genes in other lactobacilli, e.g. *L. plantarum*[24] or *L. casei* ATCC 334 [25] in connection with the broad range of substrate selectivity described for those enzymes stresses their key function of NAD^+^ regeneration. Pyruvate can be produced by *L. sanfranciscensis* from a number of substrates. Besides the usual formation from sugars and gluconate via the phosphoketolase pathway, pyruvate can be generated from asparagine and alanine via transamination and from malate catalysed by malate dehydrogenase (LSA_02100, EC 1.1.1.38).

### Formation of exopolysaccharides (EPS)

Formation of EPS is a trait often found in lactic acid bacteria [26]. Heterofermentative lactobacilli occurring in sourdough mostly synthesize glucan or fructan homopolymers. These are formed from sucrose by secreted or cell-anchored glucosyltransferases, which convert the sucrose into high-molecular-weight polymers, with the concomitant release of the respective hexose.

In TMW 1.1304 two genes encode respective glucosytransferases, both carrying a LPXTG sortase recognition motif. A plasmid encoded dextransucrase (LSA_2p00510) with a best protein match (85%) to a dextransucrase of *L. reuteri* JCM 1112 is obviously not active due to the lack of ca. 500 aa residues at the N-terminus and an atypical small molecular weight. A levansucrase (LSA_LSA_02160) was found to be identical toa levansucrase previously described by Tieking *et al*. from *L. sanfranciscensis* TMW 1.392 [27]. Interestingly, a 48 aa residue deletion corresponding to 4 direct repeats (PVNPSQPTTPAK) in the PXX motif of the C-terminal cell wall anchor is observed.

The production of EPS by TMW 1.1304 can be demonstrated by growing the strain on mMRS containing 80 g l^−1^ sucrose. To analyze the type of the polymer, the EPS was precipitated by adding two volumes of ethanol (99%) and incubation at 4°C for 18 h. EPS was hydrolyzed with perchloric acid at 100°C for 3 h and sugar monomers were analyzed with HPLC as described by Waldherr *et al*, 2008 [28]. Under these conditions the EPS produced by TMW 1.1304 was demonstrated to consist of fructose, indicating that EPS is a high molecular fructan and the levansucrase is functional.

Besides their role for bacterial metabolism, for which protective functions and an altered metabolite profile as a result of alternative use of electron acceptors are discussed, exopolysaccharides impact on crumb structure and shelf life of sourdough breads. Fructans and fructooligosaccharides likely produced by *L. sanfranciscensis* TMW 1.304 may serve further functional aspects in nutrition and medicine [29].

### Amino acid metabolism

*In silico* analyses of the genome of *L. sanfranciscensis* TMW 1.1304 indicate the potential to synthesize *de novo* four amino acids (alanine from pyruvate, aspartate from oxaloacetate, glutamate and glutamine. L-alanine can be converted into L-cysteine using a cysteine desulfurase (EC 2.8.1.7, LSA_0990), while arginine, lysine and asparagine result from conversion pathways of L-aspartate. Therefore, *L. sanfranciscensis* is auxotroph for the remaining 12 amino acids (Table 2). As concentrations in wheat of aspartate, asparagine and glutamate are low, preservation of the biosynthetic pathways for these suggests adaptation to the sourdough environment.

**Table 2:** Comparison of abundance of cytoplasmatic permeases in relation to genome size and predicted auxotrophy for amino acids between different LAB

Organism	Cytoplasmatic aminopeptidases	Genome size [Mbp]	Auxotrophic for amino acids	Reference
*L. plantarum* WCFS1	19	3.31	3	[24]
*L. sanfranciscensis* TMW 1.1304	20	1.29	12	This work
*L. acidophilus* NCFM	20	1.99	14	[31]
*L. helveticus* DPC 4571	24	2.08	16	[60]
*L. johnsonii* NC 533	25	1.99	20	[61]
*L. casei* ATCC 334	27	3.08	3	[62]
*L. gasseri* ATCC 33323	29	1.89	17	[30]

### Purine and pyrimidine biosynthesis

The enzyme required to generate 5-phosphoribosyl-1-pyrophosphate (PRPP) from the phosphoketolase pathway intermediate ribulose-5-phosphate (EC 5.3.1.6/ LSA_04470 and EC 2.7.6.1/ LSA_04050; LSA_09930) are present in *L. sanfranciscensis.* As described for *L. gasseri*[30] six of the subsequent nine enzymes required to generate IMP from PRPP seem to be absent in *L. sanfranciscensis.* However, guanosine and adenosine as well as the corresponding nucleotides could be generated from IMP.

Although all genes necessary for the *de novo* synthesis of pyrimidines are present, *L. sanfranciscensis* is presumably auxotrophic for pyrimidines. The gene for dihydroorotase (EC 3.5.2.3; LSA_05890, LSA_05900), one of the five enzymes needed to generate UMP from carbamoyl-phosphate seems to be inactive due to a frameshift. Besides the dihydroorotase gene no additional pseudogenes are present in the pyrimidine metabolism of *L. sanfranciscensis.*

### Cofactors

Similar to other lactobacilli, *L. sanfranciscensis* appears unable to synthesize most cofactors and vitamins like folate, thiamine, riboflavin, vitamine B6, nicotinate and nicotinamide. *In silico* analysis predicts that this organism can utilize both nicotinate and nicotinamide to generate NAD. However, this is only possible as two of the key enzymes, nicotinamidase (Ec 3.5.1.19) and nicotinate phosphoribosyltransferase (EC 2.4.2.11) are encoded by the plasmid pLS2 ( LSA_2p00220, LSA_2900230).

Although only one gene involved in cobalamine synthesis (cobyrinic acid a, c-diamide synthase, EC 6.3.5.11; LSA_2900630) was encoded by the sequenced strain *L. sanfranciscensis* TMW1.1304, growth experiments showed that 8 of 11 *L. sanfranciscensis* strains tested were able to grow on vitamin B12 free media (Difco®) indicating that those strains were able to synthezise cobalamine *de novo*.

### Proteolytic system

The predicted auxotrophy for 12 amino acids for *L. sanfranciscensis* was consistent with the presence of a large number of peptidases, proteases and transport systems for amino acids and peptides (Additional file 6). A complex proteolytic system ensures not only the supply with essential amino acids but also likely provides *L. sanfranciscensis* with a selective advantage in its protein-rich environment as acquisition of amino acids from the environment is energetically more favourable than *de novo* synthesis [10].

The absence of an extracellular protease (*prt*) gene in the genome of *L. sanfranciscensis* reflects its high adaptation to and the associated dependency on sourdough. In contrast, dairy lactobacilli with comparable auxotrophy for amino acids like *L. helveticus* or *L. acidophilus* encode *prt* genes for proteinase production as milk only has low proteolytic activity and therefore degradation of casein to oligopeptides is a prerequisite for the growth of lactic acid bacteria in milk [31,32].

Peptides and amino acids present in the sourdough environment are internalized by peptide transporters and amino acid permeases. The *L. sanfranciscensis* genome encodes a di-/tripeptide transporter *dtpT* (LSA_04370) and a complete oligopeptide transport system Opp. All five genes of the Opp system (*oppD*, *oppF*, *oppB*, *oppC*, *oppA*) are organized in an operon (LSA_0280-LSA_0320). In addition to several amino acid transporters with unknown specificity, ABC transporters for glutamine (*glnHMPQ*, LSA_13380-13410), methionine (LSA_12550-12560, LSA_0940, LSA_08450) and cystine (*tcyABC*, LSA_01990, LSA_8550, LSA_8540, LSA_10490) are predicted. Additionally a lysine-specific permease (LSA_11550), a serine-threonine antiporter (*steT*, LSA_03230), an arginine-ornithine antiporter (*arcD*, LSA_12260), a choline-glycine betaine transporter (LSA_11780) and a γ-aminobutyrate permease (LSA_8760) are present.

*L. sanfranciscensis* has 20 genes encoding cytoplasmatic peptidases of different specificity to hydrolyze incorporated peptides into free amino acids. (Additional file 6). Many of the genes were described in *L. sanfranciscensis* previously [33], but the genome sequence included novel genes with homology to the *pepB*, *pepD*, *pepE*, *pepM*, *pepO*, *pepQ*, and *pepV*. Compared to other LAB no correlation between amino acid auxotrophy and content of cytoplasmatic peptidases can be observed.

### Regulators

Based on the presence of conserved functional domains 2 two-component regulatory systems and 38 transcriptional regulators including five pseudogenes were predicted (Additional files 7 and 8). Compared to *L. acidophilus* NCFM that harbours 9 two-component regulatory systems, this is a quite low number of genes involved in gene regulation and might reflect adaptation to a stable and nutrient-rich environment, where less adaptive regulation is required [34][30]. Like for other sequenced lactobacilli the numerically predominant regulatory protein families are repressors, i. e. MarR (five members), AcrR (four members) and MerR (four members). Besides *cspA* and the 2 two-component regulatory systems only two transcriptional activators both belonging to the LysR family were predicted. *L. sanfranciscensis* has three genes encoding for sigma factors. Besides the primary sigma-factor *rpoD* (LSA_7720) two genes for *rpoE* (LSA_03460 and LSA_4550), a sigma factor involved in high temperature and oxidative stress response are present.

### Phage defense/restriction/modification systems

CRISPR loci play a critical role in the adaptation and persistence of a microbial host in a particular ecosystem. The observed similarity between spacers and phage or plasmid sequences has led to the hypothesis that CRISPRs may provide resistance against foreign DNA determinants [35-39]. Using CRISPRFinder [36], a web tool to identify clustered regularly interspaced short palindromic repeats (CRISPR) we identified two CRISPR loci. A chromosomally located CRISPR/cas system consists of three cas genes followed by two 29 bp CRISPR spacers. Repeat length (36 bp) and sequence similarity indicates its belonging to the Lsal1 family. A plasmid-located CRISPR/cas system consists of 5 cas genes and a CRISPR with 14 spacers, where the 28 bp repeats are separated from the cas genes by an IS607-family transposase gene. Repeat size (29bp) and sequence as well as spacer size (32/33bp) are identical to L. *brevis* ATCC 367 CRISPR Ldbu1-family [40]. Only one other plasmid-encoded CRISPR/cas system was identified on the *Enterococcus faecium* pHT beta plasmid [41]. The repeat number in *L. sanfranciscensis* is below the average number of repeats per locus of 19.5 found in other LAB [40].

Very few phages of lactobacilli have been isolated from sourdough samples [42,43] and only one phage active on *L. sanfranciscensis* was described [44]. BLAST analysis of spacer sequences resulted in the identification of only two significant hits for plasmid-encoded spacer 14 with 100 % similarity to *Lactococcus lactis* plasmid pEW104 (AF097471) and for the chromosomal spacer 2 with 93 % to *L. plantarum* plasmid pLTK2 (AB024514). No hit was found for a known phage sequence. While phage infections and spread in sourdough cultures may be hampered by the solid texture of the fermentative mass in batch systems the presence of CRISPR/cas may generally account for genetic stability of this strain in the sourdough environment making it a stable element over decades of fermentation.

### Mobile elements

The presence of 111 transposases (including 25 pseudogenes) in IS elements in five different IS element families ( IS3, IS30, ISL3, IS200/605, IS256) represent 7.7% of the ORFs found and are, as a result of the small chromosome, found in higher proportion than in other lactobacilli. Thus, an idea of facilitated niche adaptation of a distinct *Lactobacillus* subpopulation by a relative increase of genome plasticity is supported.

### Stress response

Among genes related to heat, cold, acid, DNA damage and starvation genes related to the capability to respond to osmotic and oxidative stress are pronounced despite the small genome, suggesting that *L. sanfranciscensis* frequently faces such stresses. Generally, tolerance to oxygen of lactic acid bacteria requires the presence of catalase and/or NADH oxidases or several thiol-active enzyme systems including the thioredoxin-thioredoxin reductase couple, the glutathione-GshR system and a cyst(e)ine uptake and metabolism. On the basis of sequence similarity beside a NADH oxidase (LSA_05610) we identified in *L. sanfranciscensis*, a glutathione reductase (LSA_2p00270), a glutaredoxin-like protein (LSA_04700), two thioredoxin reductases (LSA_02530, LSA_05170), a putative thioredoxin peroxidase (LSA_09790), three thioredoxin-like proteins (LSA_08950, LSA_02610, LSA_06080) and a cyst(e)ine transport protein (LSA_08550). It was previously reported that a glutathione reductase negative mutant strain of *L. sanfranciscensis* DSM20451^T^ lost oxygen tolerance and exhibited a strongly decreased aerobic growth rate compared to either the growth rate under anaerobic conditions or that of the wild-type strain. Moreover aerobic growth was restored by the addition of cysteine [45]. In the majority of organisms glutathione is synthesized by the sequential action of γ-glutamylcysteine synthetase and glutathione synthetase, encoded by *gshA* and *gshB*, respectively. The genome of *L. sanfranciscensis* contain no homolog of these two enzymes indicating that glutathione is probably to be imported from the medium. Actually, in the traditional backslopping sourdough process is a solid state fermentation with varying water activities and repeated mixing procedures frequently introduce oxygen. However, oxygen is readily used as electron acceptor in the reaction of NADH oxidase II, which directly produces water.

### Bacteriocins

The production of inhibitory substances by sourdough LAB could provide another selective advantage for the producer strains [46]. Bacteriocins so far discovered from sourdough LAB and include the bacteriocins bavaricin A [47], plantaricin ST31 [48] and the bacteriocin-like inhibitory substance *L. sanfranciscensis* C57.[49].

No functionally active bacteriocin genes are found in the genome. Only two truncated genes sharing 100% with *papA* encoding pediocin were found. Thus, bacteriocin production cannot be the reason for the long term competitiveness of this bacterium in sourdough.

### Plasmids and plasmid encoded traits

Two plasmids pLS1 and pLS2 were present in strain TMW 1.1304. While plasmid-encoded traits for lactobacilli frequently include genes for sugar metabolism plasmids pLS1 and pLS2 harbour genes involved in nucleotide/NADH metabolism and are further characterized by the presence of many orfs encoding transposases.

The total DNA sequence of pLS1 consists of 58,739 bp with a GC content of 37.6 % encoding 59 orfs. Two genes encoding Rep B (replication associated replication protein) and RepA are homologous to the corresponding genes of the *L. brevis* plasmid pLB925A04 that is a theta type replication plasmid of the pAMβ-1-family [50]. A truncated dextransucrase (LSA_2p00510) is discussed above. PLS1 contains a CRISPR/cas locus further supporting the importance of this function even at an enhanced copy number (see above).

The total DNA sequence of pLS2 consists of 18,715 bp with a GC content of 36.1 % and encodes 19 orfs. The replication protein RepB shows 80% similarity to a RepB protein of *Enterococcus faecium* E1636 (EMBL EFF23229.1) and 40 % to a RepB of plasmid pLTK13 (EMBL BAG67041), a rolling circle replicating plasmid of *L. plantarum* L137. Replication of the lagging strand of RC plasmids initiates from their single-strand origins (SSOs). SSOs have a high potential for intrastrand pairing and based on their secondary structures, several types of SSOs have been identified [51,52]. PLS2 contains a palindromic region (position 21–58) whose secondary structure is similar to the ssoA-type origin. A restriction / modification system consists of a cytosine-specific methyltransferase followed by a restriction endonuclease gene similar to the McrBC restriction endonuclease system of *Rhodobacter capsulatus* ATCC BAA-309 (EMBL ADE85042 .1).

## Material and methods

### Strain selection, strain purification and DNA isolation

For sequencing a strain without any laboratory transfers was selected to ensure sequencing of a truly sourdough adapted clone. Therefore, the strain was isolated on mMRS [53] at 30°C from “Böcker Reinzuchtsauer”, a rye sourdough starter, which is now propagated for 100 years in the same tradition, and only propagated on laboratory media to obtain enough DNA for sequencing. Dilutions of a sourdough sample were spread directly on mMRS agar plates. Plates were incubated under anaerobic conditions at 30 °C for 3–5 days. Genomic DNA was isolated with the EZNA DNA reagent set (Omega Bio-Tek) according the provided protocol for Gram positive bacteria.

### Sequencing strategy

Sequencing was done in a combined Sanger/454-pyrosequencing approach. 454 sequencing resulted in 187,929 reads with an average read length of 250 nucleotides giving ~45 Mbp sequencing data corresponding to a 33-fold coverage. In addition 10,000 genomic fragments with typically 3kb to 5 kb inserts were cloned into the TOPO TA vector (Quiagen, Hilden) and sequenced on an ABI 3730 capillar sequencer from both ends. The ABI sequences resulted in 19.569 reads corresponding to an additional coverage of 13 fold. Remaining gaps were closed by sequences generated on gap-spanning PCR products by an ABI 3730 capillary sequencer. The overall quality was set to a minimum confidence of PHRED 45 for the complete genome.

This genome project has been deposited in the European Molecular Biology Laboratory (EMBL)/Gen- Bank under the accession numbers CP002461 (chromosome), CP002462 (pLS1) and CP002463 (pLS2). The version described in this paper is the first version. Prediction of protein encoding sequences and open reading frames (ORFs) were initially accomplished with PEDANT software suite [54]. The PEDANT genome database provides exhaustive annotation of nearly 3000 publicly available eukaryotic, eubacterial, archaeal and viral genomes. Gene prediction was performed with GenMark 2.8 [55]and Glimmer 3.0 [56]as implemented in the Pedant software suite. All orf predictions were verified and modified by a blasting orfs to NCBI nrdb. Additionally, the predicted start codons of all ORFs were inspected manually using the Artemis program [57]. Clustered regularly interspaced short palindromic repeats (CRISPR) were identified with the web tool CRISPRFinder [36].

### Phylogenetic tree

A phylogenetic tree on the basis of a multiple 16S rDNA alignment based similarity matrix was constructed by the neighbour-joining method [58] using the software package Bionumerics v6.5 (Applied Maths, Belgium). Unknown bases were discarded for the analyses. Bootstrapping analysis was undertaken to test the statistical reliability of the topology of the neighbour-joining tree using 100 bootstrap resamplings of the data

### Exopolysaccharide analysis

For production of EPS strain TMW 1.1304 was grown on mMRS containing 80 g l−1 of sucrose at 30°C for 24 h. To analyze the type of the polymer the EPS was precipitated by adding two volumes of ethanol (99%) and incubation at 4°C for 18 h. EPS was hydrolyzed with perchloric acid at 100°C for 3 h and sugar monomers were analyzed with HPLC as described by Waldherr *et al*. 2008 [28].

## Competeng interests

The authors declare that they have no competing interests.

## Supplementary Material

Additional file 1 Protein length distribution and average orf length of L. sanfranciscensis TMW 1.1304 compared to other lactobacilli genomes. Data were extracted from the PEDANT 3 database (Walter et al. 2009) Click here for file

Additional file 2 List of genomes used for the analysis of the rRNA operon density. Click here for file

Additional file 3 List of genomes with rRNA gene densities above 2.9 per Mbp. Click here for file

Additional file 4 List of Lactobacillus sanfranciscensis genes which are translationally optimized with respect to codon usage bias. Click here for file

Additional file 5 In silico analysis of the genome of L. sanfranciscensis TMW 1.1304 for ORFs putatively involved in the utilization of different carbon sources Click here for file

Additional file 6 Summary of the L. sanfranciscensis cytoplasmatic proteases and peptidases, and their cleavage specificities, gene names and relative abundance in the genome. The ' | ' indicates the cleavage site. Click here for file

Additional file 7 Transcriptional regulators witin the genome of Lactobacillus sanfranciscensis TMW 1.1304 Click here for file

Additional file 8 Presence of genes for transcriptional regulators and two-component regulatory systems in different lactobacilli genomes Click here for file

## References

[B1] Vogel RF Gänzle MG Bread is an essential part of human nutrition and culture Food Microbiology 2009 26 665 671 10.1016/j.fm.2009.07.013 19747598

[B2] Kline L Sugihara TF Microorganisms of the San Francisco sour dough bread process. II. Isolation and characterization of undescribed bacterial species responsible for the souring activity Appl Microbiol 1971 21 459 465 5553285 10.1128/am.21.3.459-465.1971 PMC377203

[B3] Weiss N Schillinger U *Lactobacillus sanfrancisco* sp. nov., nom. rev System Appl Microbiol 1984 5 230 232

[B4] Trüper H-G L DC Taxonomic note: necessary correction of specific epithets formed as substantives (nouns) “in apposition Int J Syst Bacteriol 1997 47 908 909 10.1099/00207713-47-3-908

[B5] Gobbetti M Corsetti A *Lactobacillus sanfrancisco* a key sourdough lactic acid bacterium: a review Food Microbiology 1997 14 175 187 10.1006/fmic.1996.0083

[B6] Vogel RF Ehrmann MA Ganzle MG Development and potential of starter lactobacilli resulting from exploration of the sourdough ecosystem Antonie Van Leeuwenhoek 2002 81 631 638 10.1023/A:1020530227192 12448759

[B7] Groeneveld WH Van Reenen CA Todorov SD DuToit M Witthuhn RC Holzapfel WH Dicks LMT Identification of lactic acid bacteria from vinegar fruit flies based on their phenotypic and genotypic characteristics Am J Enol Vitic 2006 57 519 525

[B8] Thiele C Gaenzle MG Vogel RF Contribution of sourdough lactobacilli, yeast, and cereal enzymes to the generation of amino acids in dough relevant for bread flavour Cereal Chemistry 2002 79 45 51 10.1094/CCHEM.2002.79.1.45

[B9] Gallo G De Angelis M McSweeney PLH Corbo MR Gobbetti M Partial purification and characterization of an X-prolyl dipeptidyl aminopeptidase from *Lactobacillus sanfranciscensis* CB1 Food Chemistry 2005 91 535 544 10.1016/j.foodchem.2004.08.047

[B10] Gobbetti M Smacchi E Corsetti A The proteolytic system of *Lactobacillus sanfrancisco* CB1: purification and characterization of a proteinase, a dipeptidase, and an aminopeptidase Appl Environ Microbiol 1996 62 3220 3226 8795211 10.1128/aem.62.9.3220-3226.1996 PMC168117

[B11] De Angelis LM Rossi J Servili M Fox PF Rollán G Gobbetti M Arginine catabolism by sourdough lactic acid bacteria: purification and characterization of the arginine deiminase pathway enzymes from *Lactobacillus sanfranciscensis* CB Applied and Environmental Microbiology 2002 68 6193 6201 10.1128/AEM.68.12.6193-6201.2002 12450844 PMC134416

[B12] Tieking M Ehrmann MA Vogel RF Ganzle MG Molecular and functional characterization of a levansucrase from the sourdough isolate *Lactobacillus sanfranciscensis* TMW 1.392 Appl Microbiol Biotechnol 2005 66 655 663 10.1007/s00253-004-1773-5 15735966

[B13] Zapparoli G Toriani S Dellaglio F Differentiation of *Lactobacillus sanfrancicensis* strains by randomly amplified polymorphic DNA and pulsed-field gel electrophoresis FEMS Microbiol Lett 1998 166 325 332 10.1111/j.1574-6968.1998.tb13908.x

[B14] Macklaim JM Gloor GB Anukam KC Cribby S Reid G At the crossroads of vaginal health and disease, the genome sequence of *Lactobacillus iners* AB-1 Proceedings of the National Academy of Sciences 2010 http://www.pnas.org/cgi/doi/10.1073/pnas.1000086107 10.1073/pnas.1000086107 PMC3063587 21059957

[B15] Tamames J Gil R Latorre A Pereto J Silva FJ Moya A The frontier between cell and organelle: genome analysis of Candidatus *Carsonella ruddii* BMC Evol Biol 2007 7 181 10.1186/1471-2148-7-181 17908294 PMC2175510

[B16] Condon C Liveris D Squires C Schwartz I Squires CL rRNA operon multiplicity in *Escherichia coli* and the physiological implications of rrn inactivation J Bacteriol 1995 177 4152 4156 7608093 10.1128/jb.177.14.4152-4156.1995 PMC177152

[B17] Klappenbach JA Dunbar JM Schmidt TM rRNA operon copy number reflects ecological strategies of bacteria Appl Environ Microbiol 2000 66 1328 1333 10.1128/AEM.66.4.1328-1333.2000 10742207 PMC91988

[B18] Andersson SGaK C G Codon preferences in free-living microorganisms Microbiol Rev 1990 54 198 210 2194095 10.1128/mr.54.2.198-210.1990 PMC372768

[B19] Sharp PM Bailes E Grocock RJ Peden JF Sockett RE Variation in the strength of selected codon usage bias among bacteria Nucleic Acids Res 2005 33 1141 1153 10.1093/nar/gki242 15728743 PMC549432

[B20] Van Mandach C Merkl R Genes optimized by evolution for accurate and fast translation encode in Archaea and Bacteria a broad and characteristic spectrum of protein functions BMC Genomics 2010 11 617 10.1186/1471-2164-11-617 21050470 PMC3091758

[B21] Stolz P Böcker G Hammes WP Vogel RF Utilization of electron acceptors by lactobacilli isolated from sourdough. I. *Lactobacillus sanfrancisco* Z Lebensm Unters Forsch 1995 201 91 96 10.1007/BF01193208

[B22] Zhang C Ganzle MG Metabolic pathway of α-ketoglutarate in *Lactobacillus sanfranciscensis* and *Lactobacillus reuteri* during sourdough fermentation Journal of Applied Microbiology 2010 109 1301 1310 10.1111/j.1365-2672.2010.04753.x 20477886

[B23] Radler F Brohl K The metabolism of several carboxylic acids by lactic acid bacteria Z Lebensm Unters Forsch 1984 179 228 231 10.1007/BF01041899 6495871

[B24] Kleerebezem M Boekhorst J van Kranenburg R Molenaar D Kuipers OP Leer R Tarchini R Peters SA Sandbrink HM Fiers MW Complete genome sequence of *Lactobacillus plantarum* WCFS1 Proc Natl Acad Sci U S A 2003 100 1990 1995 10.1073/pnas.0337704100 12566566 PMC149946

[B25] Rico J Yebra MJ Perez-Martinez G Deutscher J Monedero V Analysis of ldh genes in *Lactobacillus casei* BL23: role on lactic acid production J Ind Microbiol Biotechnol 2008 35 579 586 10.1007/s10295-008-0319-8 18231816

[B26] Waldherr F Vogel RF Ulrich M Commercial Exploitation of Homo-exopolysaccharides in Non-dairy Food Systems Bacterial Exopolysaccharides - current innovations and future trends 2009 Caister Academic Press

[B27] Tieking M Kuhnl W Ganzle MG Evidence for formation of heterooligosaccharides by *Lactobacillus sanfranciscensis* during growth in wheat sourdough J Agric Food Chem 2005 53 2456 2461 10.1021/jf048307v 15796579

[B28] Waldherr FW Meissner D Vogel RF Genetic and functional characterization of Lactobacillus panis levansucrase Arch Microbiol 2008 190 497 505 10.1007/s00203-008-0404-4 18607568

[B29] Korakli M Vogel RF Structure/function relationship of homopolysaccharide producing glycansucrases and therapeutic potential of their synthesised glycans Appl Microbiol Biotechnol 2006 71 790 803 10.1007/s00253-006-0469-4 16724190

[B30] Azcarate-Peril MA Altermann E Goh YJ Tallon R Sanozky-Dawes RB Pfeiler EA O'Flaherty S Buck BL Dobson A Duong T Analysis of the genome sequence of *Lactobacillus gasseri* ATCC 33323 reveals the molecular basis of an autochthonous intestinal organism Appl Environ Microbiol 2008 74 4610 4625 10.1128/AEM.00054-08 18539810 PMC2519322

[B31] Altermann E Russell WM Azcarate-Peril MA Barrangou R Buck BL McAuliffe O Souther N Dobson A Duong T Callanan M Complete genome sequence of the probiotic lactic acid bacterium *Lactobacillus acidophilus* NCFM Proc Natl Acad Sci U S A 2005 102 3906 3912 10.1073/pnas.0409188102 15671160 PMC554803

[B32] Pastar I Begovic J Lozo J Topisirovic L Golic N Casitone-dependent transcriptional regulation of the prtP and prtM genes in the natural isolate *Lactobacillus paracasei* subsp. *paracasei* Folia Microbiol (Praha) 2007 52 577 584 10.1007/BF02932186 18450219

[B33] Vermeulen N Pavlovic M Ehrmann MA Ganzle MG Vogel RF Functional characterization of the proteolytic system of *Lactobacillus sanfranciscensis* DSM 20451T during growth in sourdough Appl Environ Microbiol 2005 71 6260 6266 10.1128/AEM.71.10.6260-6266.2005 16204547 PMC1266010

[B34] Stock AM Robinson VL Goudreau PN Two-component signal transduction Annu Rev Biochem 2000 69 183 215 10.1146/annurev.biochem.69.1.183 10966457

[B35] Bolotin A Quinquis B Sorokin A Ehrlich SD Clustered regularly interspaced short palindrome repeats (CRISPRs) have spacers of extrachromosomal origin Microbiology 2005 151 2551 2561 10.1099/mic.0.28048-0 16079334

[B36] Grissa I Vergnaud G Pourcel C CRISPRFinder: a web tool to identify clustered regularly interspaced short palindromic repeats Nucleic Acids Res 2007 35 52 57 10.1093/nar/gkm360 PMC1933234 17537822

[B37] Horvath P Romero DA Coute-Monvoisin AC Richards M Deveau H Moineau S Boyaval P Fremaux C Barrangou R Diversity, activity, and evolution of CRISPR loci in *Streptococcus thermophilus* J Bacteriol 2008 190 1401 1412 10.1128/JB.01415-07 18065539 PMC2238196

[B38] Makarova KS Grishin NV Shabalina SA Wolf YI Koonin EV A putative RNA-interference-based immune system in prokaryotes: computational analysis of the predicted enzymatic machinery, functional analogies with eukaryotic RNAi, and hypothetical mechanisms of action Biol Direct 2006 1 7 10.1186/1745-6150-1-7 16545108 PMC1462988

[B39] Mojica FJ Diez-Villasenor C Garcia-Martinez J Soria E Intervening sequences of regularly spaced prokaryotic repeats derive from foreign genetic elements J Mol Evol 2005 60 174 182 10.1007/s00239-004-0046-3 15791728

[B40] Horvath P Coute-Monvoisin AC Romero DA Boyaval P Fremaux C Barrangou R Comparative analysis of CRISPR loci in lactic acid bacteria genomes Int J Food Microbiol 2009 131 62 70 10.1016/j.ijfoodmicro.2008.05.030 18635282

[B41] Tomita H Ike Y Genetic analysis of transfer-related regions of the vancomycin resistance *Enterococcus* conjugative plasmid pHTbeta: identification of oriT and a putative relaxase gene J Bacteriol 2005 187 7727 7737 10.1128/JB.187.22.7727-7737.2005 16267297 PMC1280310

[B42] Foschino R Perrone F Galli A Characterization of two virulent *Lactobacillus fermentum* bacteriophages isolated from sour dough J Appl Bact 1995 79 677 683

[B43] Foschino R Galli A Pagani A Ottogalli G Isolation and characterization of bacteriophages active on heterofermentative lactobacilli in sourdoughs Microbiol Alim Nutr 1996 14 15 22

[B44] Foschino R Venturelli E Picozzi C Isolation and characterization of a virulent *Lactobacillus sanfranciscensis* bacteriophage and its impact on microbial population in sourdough Curr Microbiol 2005 51 413 418 10.1007/s00284-005-0122-y 16235023

[B45] Jaensch A Korakli M Vogel RF Ganzle MG Glutathione reductase from *Lactobacillus sanfranciscensis* DSM20451^T^: contribution to oxygen tolerance and thiol exchange reactions in wheat sourdoughs Appllied and Environmental Microbiology 2007 73 4469 4476 10.1128/AEM.02322-06 PMC1932818 17496130

[B46] Messens W DeVuyst L Inhibitory substances produced by Lactobacilli isolated from sourdoughs--a review Int J Food Microbiol 2002 72 31 43 10.1016/S0168-1605(01)00611-0 11843411

[B47] Larsen AG Vogensen FK Josephsen J Antimicrobial activity of lactic acid bacteria isolated from sour doughs: purification and characterization of bavaricin A, a bacteriocin produced by *Lactobacillus bavaricus* MI401 J Appl Bacteriol 1993 75 113 122 8407671 10.1111/j.1365-2672.1993.tb02755.x

[B48] Todorov S Onno B Sorokine O Chobert JM Ivanova I Dousset X Detection and characterization of a novel antibacterial substance produced by *Lactobacillus plantarum* ST 31 isolated from sourdough Int J Food Microbiol 1999 48 167 177 10.1016/S0168-1605(99)00048-3 10443536

[B49] Corsetti A Gobbetti M Smacchi E Antibacterial activity of sourdough lactic acid bacteria: isolation of a bacteriocin-like inhibitory substance from *Lactobacillus sanfrancisco* Food microbiology 1996 13 447 456 10.1006/fmic.1996.0051

[B50] Wada T Noda M Kashiwabara F Jeon HJ Shirakawa A Yabu H Matoba Y Kumagai T Sugiyama M Characterization of four plasmids harboured in a *Lactobacillus brevis* strain encoding a novel bacteriocin, brevicin 925A, and construction of a shuttle vector for lactic acid bacteria and *Escherichia coli* Microbiology 2009 155 1726 1737 10.1099/mic.0.022871-0 19372160

[B51] Khan SA Rolling-circle replication of bacterial plasmids Microbiol Mol Biol Rev 1997 61 442 455 9409148 10.1128/mmbr.61.4.442-455.1997 PMC232620

[B52] Khan SA Plasmid rolling-circle replication: recent developments Mol Microbiol 2000 37 477 484 10931341 10.1046/j.1365-2958.2000.02001.x

[B53] Stolz P Hammes WP Vogel RF Maltose-phosphorylase and hexokinase activity in lactobacilli from traditionally prepared sourdoughs Adv Food Sci 1996 18 1 6

[B54] Walter MC Rattei T Arnold R Guldener U Munsterkotter M Nenova K Kastenmuller G Tischler P Wolling A Volz A PEDANT covers all complete RefSeq genomes Nucleic Acids Res 2009 37 D408 411 10.1093/nar/gkn749 18940859 PMC2686588

[B55] Borodovsky M Mills R Besemer J Lomsadze A Prokaryotic gene prediction using GeneMark and GeneMark.hmm Curr Protoc Bioinformatics 2003 5 Chapter 4:Unit4 10.1002/0471250953.bi0405s01 18428700

[B56] Delcher AL Bratke KA Powers EC Salzberg SL Identifying bacterial genes and endosymbiont DNA with Glimmer Bioinformatics 2007 23 673 679 10.1093/bioinformatics/btm009 17237039 PMC2387122

[B57] Rutherford K Parkhill J Crook J Horsnell T Rice P Rajandream MA Barrell B Artemis: sequence visualization and annotation Bioinformatics 2000 16 944 945 10.1093/bioinformatics/16.10.944 11120685

[B58] Saitou N Nei M The neighbor-joining method: a new method for reconstructing phylogenetic trees Molecular Biology Evolution 1987 4 406 425 10.1093/oxfordjournals.molbev.a040454 3447015

[B59] Morita H Toh H Fukuda S Horikawa H Oshima K Suzuki T Murakami M Hisamatsu S Kato Y Takizawa T Comparative genome analysis of *Lactobacillus reuteri* and *Lactobacillus fermentum* reveal a genomic island for reuterin and cobalamin production DNA Res 2008 15 151 161 10.1093/dnares/dsn009 18487258 PMC2650639

[B60] Callanan M Kaleta P O'Callaghan J O'Sullivan O Jordan K McAuliffe O Sangrador-Vegas A Slattery L Fitzgerald GF Beresford T Ross RP Genome sequence of *Lactobacillus helveticus*, an organism distinguished by selective gene loss and insertion sequence element expansion J Bacteriol 2008 190 727 735 10.1128/JB.01295-07 17993529 PMC2223680

[B61] Pridmore RD Berger B Desiere F Vilanova D Barretto C Pittet AC Zwahlen MC Rouvet M Altermann E Barrangou R The genome sequence of the probiotic intestinal bacterium *Lactobacillus johnsonii* NCC 533 Proc Natl Acad Sci U S A 2004 101 2512 2517 10.1073/pnas.0307327101 14983040 PMC356981

[B62] Cai H Thompson R Budinich MF Broadbent JR Steele JL Genome sequence and comparative genome analysis of *Lactobacillus casei*: insights into their niche-associated evolution Genome Biol Evol 2009 1 239 257 20333194 10.1093/gbe/evp019 PMC2817414

