# Supplementary material for: Genomic analysis reveals Lactobacillus sanfranciscensis as stable element in traditional sourdoughs
Source: Microb Cell Fact. 2011 Aug 30;10(Suppl 1):S6. doi: 10.1186/1475-2859-10-S1-S6 (PMC3231932; doi:10.1186/1475-2859-10-S1-S6)
Supplement: Additional file 1 — Protein length distribution and average orf length of L. sanfranciscensis TMW 1.1304 compared to other lactobacilli genomes. Data were extracted from the PEDANT 3 database (Walter et al. 2009) [file 1475-2859-10-S1-S6-S1.docx]

# Table S1. Protein length distribution and average orf length of *L. sanfranciscensis* TMW 1.1304 compared to other lactobacilli genomes. Data were extracted from the PEDANT 3 database (Walter et al. 2009)

|  | *L. sanfranciscensis* TMW 1.1304 | *L. acidophilus* NCFM | *L. brevis* ATCC 367 | *L. plantarum* WCFS1 | *L. sakei* 23K | *L. johnsonii* NCC 533 | *L. casei* ATCC 334 | *L. helveticus* DPC 4571 | *L. reuteri* JCM 1112^T^ |
| --- | --- | --- | --- | --- | --- | --- | --- | --- | --- |
| Genome size [nt]^1^ | 1,298,316 | 1,993,564 | 2,291,220 | 3,308,274 | 1,884,661 | 1,992,676 | 2,895,264 | 2,080,931 | 2,039,414 |
| orfs | 1355 | 1864 | 2185 | 3052 | 1879 | 1821 | 2751 | 1610 | 1820 |
| average orf length [aa] | 835 | 940 | 883 | 917 | 871 | 977 | 858 | 953 | 937 |
| Coding density [%] | 87,1 | 87,9 | 84.2 | 84,3 | 86,8 | 89.2 | 80.5 | 73,7 | 83.6 |
| protein length distribution [%] | | | | | | | | | |
| <50 aa | 2,6 | 2.8 | 1.6 | 0.8 | 2.6 | 0.4 | 1.6 | 0.4 | 0.5 |
| 50-149 aa | 26,2 | 18.7 | 22.5 | 21,7 | 21.2 | 19.8 | 22.5 | 16.4 | 17.3 |
| 150-299 aa | 34.7 | 36.6 | 35.4 | 36.2 | 37.5 | 37.7 | 35.4 | 37.5 | 38.2 |
| 300-999 aa | 35.7 | 40.5 | 39.8 | 40.2 | 38.1 | 40.6 | 39.8 | 44.4 | 42.9 |
| 1000-3000 aa | 0.8 | 1.3 | 0.7 | 1 | 0.7 | 1.3 | 0.7 | 1 | 1 |
| >3000 aa | 0 | 0.1 | 0 | 0.1 | 0 | 0.2 | 0 | 0 | 0 |

^1^, without plasmids
