# Supplementary material for: Genomic analysis reveals Lactobacillus sanfranciscensis as stable element in traditional sourdoughs
Source: Microb Cell Fact. 2011 Aug 30;10(Suppl 1):S6. doi: 10.1186/1475-2859-10-S1-S6 (PMC3231932; doi:10.1186/1475-2859-10-S1-S6)
Supplement: Additional file 5 — In silico analysis of the genome of L. sanfranciscensis TMW 1.1304 for ORFs putatively involved in the utilization of different carbon sources [file 1475-2859-10-S1-S6-S5.docx]

Table S5. *In silico* analysis of the genome of *L. sanfranciscensis* TMW 1.1304 for ORFs putatively involved in the utilization of different carbon sources

| **Sugar** | **gene** | | **No. of genes** | **ORF** |  |
| --- | --- | --- | --- | --- | --- |
| Maltose | Maltose phosphorylase | | 1 | LSA_01510 |  |
|  | phosphoglucomutase | | 2 | LSA_12330;  LSA_01530 (putative) |  |
|  | alpha-glucosidase ( pseudogene) | | 1 | LSA_05800 |  |
|  |  | |  |  |  |
| Fructose | fructose permease , | | 1 | LSA_2810 |  |
|  | fructokinase | | 1 | LSA_05780 |  |
|  | phosphoglucose isomerase | | 1 | LSA_05790 |  |
| Ribose | ribokinase | | 1 | LSA_02830 |  |
|  | phosphoglucomutase | | 1 | LSA_12330  LSA_01530 (putative) |  |
|  | Ribose-5-phosphate isomerase | | 1 | LSA_04470 |  |
|  | Ribose-phosphate diphosphokinase | | 1 | LSA_04050 |  |
|  | phosphogluconate dehydrogenase | | 1 | LSA_00660 |  |
|  | Ribose uptake protein rbsU | | 2 | LSA_04770, LSA02730 |  |
| Gluconate | H+/gluconate symporter (pseudogene) | | 1 | LSA_2670 |  |
|  | gluconate kinase | | 1 | LSA_10640 |  |
| Other | oligo-1,6-glucosidase | | 2 | LSA_05810, LSA_01770 |  |
|  | Galactose mutarotase *galM* | | 1 | LSA_01520 |  |
|  | D-xylose:proton symporter (pseudogene) | 1 | | LSA_12950 |  |
|  | arabinose efflux permease | 2 | | LSA_01450  LSA_ 01460(pseudogene) |  |
|  | aldose-1-epimerase | 2 | | LSA_03870, LSA_07410 |  |
|  | alpha-glucosidase | | 1 | LSA_05800) |  |
