# Supplementary material for: Genomic analysis reveals Lactobacillus sanfranciscensis as stable element in traditional sourdoughs
Source: Microb Cell Fact. 2011 Aug 30;10(Suppl 1):S6. doi: 10.1186/1475-2859-10-S1-S6 (PMC3231932; doi:10.1186/1475-2859-10-S1-S6)
Supplement: Additional file 6 — Summary of the L. sanfranciscensis cytoplasmatic proteases and peptidases, and their cleavage specificities, gene names and relative abundance in the genome. The ' | ' indicates the cleavage site. [file 1475-2859-10-S1-S6-S6.docx]

**Table S6.** Summary of the *L. sanfranciscensis* cytoplasmatic proteases and peptidases, and their cleavage specificities, gene names and relative abundance in the genome The ' | ' indicates the cleavage site.

| \| **peptidase** \| \| --- \| | **specificity** | \| **gene** \| \| --- \| | **Number of genes (n=)** | **Locus number** |
| --- | --- | --- | --- | --- | --- | --- |
|  |  |  |  |  |
| **Aminopeptidases** |  |  |  |  |
| aminopeptidase N | X \| (X)n | *pepN* | 1 | L[SA_00330](http://pedant.gsf.de:8045/pedant3htmlview/pedant3view?Db=p3_i12_t1625_Lac_sanf_v3&Method=ReportGene&GeneticelemID=33) |
| aminopeptidase E | X \| (X)n | *pepE* | 1 | [LSA_13430](http://pedant.gsf.de:8045/pedant3htmlview/pedant3view?Db=p3_i12_t1625_Lac_sanf_v3&Method=ReportGene&GeneticelemID=1386) |
| aminopeptidase C | X \| (X)n | *pepC* | 1 | [LSA_04460](http://pedant.gsf.de:8045/pedant3htmlview/pedant3view?Db=p3_i12_t1625_Lac_sanf_v3&Method=ReportGene&GeneticelemID=307) |
| aminopeptidase M (Met) | Met \| (X)n | *pepM* | 1 | LSA_02870 |
|  |  |  |  |  |
| **Endopeptidases** |  |  |  |  |
| endopeptidase | (X)n \| (X)n | *pepO;* | 1 | [LSA_02950](http://pedant.gsf.de:8045/pedant3htmlview/pedant3view?Db=p3_i12_t1625_Lac_sanf_v3&Method=ReportGene&GeneticelemID=307) |
| endopeptidase (glycopeptidase) | (X)n \| (X)n |  | 2 | LSA_04870; LSA_04890 |
| endopeptidase | (X)n \| (X)n |  | 2 | LSA_08250; LSA_09800 |
| **Oligo-/Tri-/di-peptidases** |  |  |  |  |
| dipeptidase A | X \| X |  | 1 | LSA_10510 |
| Prolyl-dipeptidase (prolinase) | X \| Pro | *pepR* | 1 | LSA_03150 |
| tripeptidase T | X \| X-X | *pepT* | 2 | [LSA_00930](http://pedant.gsf.de:8045/pedant3htmlview/pedant3view?Db=p3_i12_t1625_Lac_sanf_v3&Method=ReportGene&GeneticelemID=99); [LSA_11660](http://pedant.gsf.de:8045/pedant3htmlview/pedant3view?Db=p3_i12_t1625_Lac_sanf_v3&Method=ReportGene&GeneticelemID=1203) |
| Xaa-Pro dipeptidase | X \| Pro | *pepQ* | 1 | LSA_05620 |
| beta-Ala-Xaa dipeptidase | X \| X | *pepV* | 1 | [LSA_08460](http://pedant.gsf.de:8045/pedant3htmlview/pedant3view?Db=p3_i12_t1625_Lac_sanf_v3&Method=ReportGene&GeneticelemID=876) |
| oligopeptidase | X \| (X)n | *pepB* | 1 | LSA_10280 |
| Xaa-Pro dipeptidyl-peptidase | X-Pro \| (X)n | *pepX* | 1 | LSA_11480 |
| dipeptidase | X \| X | *pepD* | 1 | LSA_06910 |
|  |  |  |  |  |
| **uncharacterized** |  |  |  |  |
| M16 family peptidase |  |  | 1 | LSA_09160 |
| S9 family peptidase |  |  | 1 | LSA_12770 |
|  | | | | |
