# Supplementary material for: Genomic analysis reveals Lactobacillus sanfranciscensis as stable element in traditional sourdoughs
Source: Microb Cell Fact. 2011 Aug 30;10(Suppl 1):S6. doi: 10.1186/1475-2859-10-S1-S6 (PMC3231932; doi:10.1186/1475-2859-10-S1-S6)
Supplement: Additional file 7 — Transcriptional regulators witin the genome of Lactobacillus sanfranciscensis TMW 1.1304 [file 1475-2859-10-S1-S6-S7.docx]

Table S7 Transcriptional regulators witin the genom of *Lactobacillus sanfranciscensis* TMW 1.1304

| **ORF** | **Annotation** | **Family** | **Closest Homology** | **Positive or Negative Regulation** |
| --- | --- | --- | --- | --- |
| LSA_01060 | Transcriptional regulator | AcrR | *Enterococcus faecalis* | *-* |
| LSA_01940 | Transcriptional regulator | AcrR | *Lactobacillus reuteri* | *-* |
| LSA_08060 | Transcriptional regulator (putative pseudogene) | AcrR | *Lactobacillus casei* | *-* |
| LSA_11880 | Transcriptional regulator | AcrR | *Pediococcus pentosaceus* | *-* |
| LSA_02250 | Transcriptional regulator | ArsR | *Lactobacillus brevis* | *-* |
| LSA_04900 | Transcriptional repressor *sdpR* | ArsR | *Streptococcus gordonii* | *-* |
| LSA_02290 | Transcriptional regulator | GntR | *Lactobacillus brevis* | *-* |
| LSA_ 04350 | HTH-type transcriptional regulator *glnR* | GlnR | *Lactobacillus ruminis* | *-* |
| LSA_04380 | Transcriptional regulator | GntR | *Lactobacillus hilgardii* | *-* |
| LSA_01490 | Transcriptional regulator (putative pseudogene) | LacI | *Leuconostoc citreum* | *-* |
| LSA_02090 | Malolactic fermentation transcriptional regulator | LysR | *Lactobacillus sakei* | *+* |
| LSA_13470 | Transcriptional regulator | LysR | *Lactobacillus brevis* | *+* |
| LSA_04430 | Membrane-bound protein *lytR* | LytR | *Lactobacillus buchneri* | Response regulator |
| LSA_12670 | Transcriptional regulator | LytR | *Lactobacillus brevis* | Response regulator |
| LSA_2p00080 | Transcriptional regulator (putative pseudogene) | LytR | *Lactobacillus brevis* | Response regulator |
| LSA_00890 | Transcriptional regulator | MarR | *Lactobacillus brevis* | *-* |
| LSA_01150 | Transcriptional regulator | MarR | *Caldicellulosiruptor saccharolyticus* | *-* |
| LSA_05940 | Transcriptional regulator (putative pseudogene) | MarR | *Lactobacillus brevis* | *-* |
| LSA_07260 | Transcriptional regulator | MarR | *Lactobacillus brevis* | *-* |
| LSA_12860 | Transcriptional regulator | MarR | *Lactobacillus salivarius* | *-* |
| LSA_13090 | Transcriptional regulator | MarR | *Lactobacillus brevis* | *-* |
| LSA_00820 | Transcriptional regulator | MerR | *Lactobacillus brevis* | *-* |
| LSA_02560 | Predicted transcriptional regulator | MerR | *Lactobacillus sakei* | *-* |
| LSA_04780 | Transcriptional regulator (putative pseudogene) | MerR | *Enterococcus faecalis* | *-* |
| LSA_ 04790 | Transcriptional regulator (putative pseudogene) | MerR | *Clostridium sp.* | *-* |
| LSA_ 06150 | Transcriptional repressor *nrdR* | NrdR | *Listeria grayi* | *-* |
| LSA_00140 | Transcriptional regulatory protein | OmpR | *Lactobacillus salivarius* | Response regulator |
| LSA_03780 | Regulatory protein v*anR* | OmpR | *Lactobacillus brevis* | Response regulator |
| LSA_12310 | Transcriptional regulator | TetR | *Lactobacillus salivarius* | *-* |
| LSA_ 07980 | Transcriptional regulator | TetR\AcrR | *Leuconostoc mesenteroides* | *-* |
| LSA_02390 | Transcriptional regulator | Xre | *Lactobacillus brevis* | *-* |
| LSA_09320 | Transcriptional regulator | Xre | *Lactobacillus hilgardii* | *-* |
| LSA_04930 | Redox-sensing transcriptional repressor *rex* |  | *Lactobacillus brevis* | *-* |
| LSA_06360 | Uncharacterized HTH-type transcriptional regulator yodB |  | *Lactobacillus fermentum* |  |
| LSA_06800 | Transcriptional regulator |  | *Lactobacillus vaginalis* |  |
| LSA_11440 | Transcriptional regulator *ctsR* |  | *Lactobacillus hilgardii* | *-* |
| LSA_ 11860 | Cold shock protein 2 *cspA* transcriptional regulator |  | *Lactobacillus hilgardii* | *+* |
| LSA_ 11870 | Cold shock protein 2 *cspA* transcriptional regulator |  | *Lactobacillus hilgardii* | *+* |
