# Supplementary material for: Genomic analysis reveals Lactobacillus sanfranciscensis as stable element in traditional sourdoughs
Source: Microb Cell Fact. 2011 Aug 30;10(Suppl 1):S6. doi: 10.1186/1475-2859-10-S1-S6 (PMC3231932; doi:10.1186/1475-2859-10-S1-S6)
Supplement: Additional file 8 — Presence of genes for transcriptional regulators and two-component regulatory systems in different lactobacilli genomes [file 1475-2859-10-S1-S6-S8.docx]

Table S8. Presence of genes for transcriptional regulators and Two-component regulatory systems in different lactobacilli genomes

| Strain | No. of transcriptional regulators | No. of Two-Component regulatory systems | Genome size (Mbp) | reference |
| --- | --- | --- | --- | --- |
| *L. bulgaricus* ATCC 11842 | 53 | 5 | 1.86 | Van de Guchte et al., 2006 |
| *L. plantarum* WSF1 | 234 | 13 | 3.31 | Kleerebezem et al., 2003 |
| *L. sanfranciscensis* TMW 1.1304 | 38 | 2 | 1.30 | This study |
| *L. acidophilus* NCFM | 96 | 9 | 1.99 | Altermann et al. 2005 |
| *L. johnsonii NCC 334* | n.d. | 9 | 1.99 | Pridmore et al., 2004 |
| *L.gasseri* ATCC 33323 | 70 | 5 | 1.89 | Peril et al., 2008 |
